# Serum immune checkpoint profiling identifies soluble CD40 as a biomarker for pancreatic cancer

**DOI:** 10.1038/s41698-023-00459-9

**Published:** 2023-10-14

**Authors:** David Digomann, Max Heiduk, Charlotte Reiche, Jessica Glück, Christoph Kahlert, Peter Mirtschink, Anna Klimova, Florian Bösch, Torsten Tonn, Jochen Gaedcke, Michael Ghadimi, Jürgen Weitz, Lena Seifert, Adrian M. Seifert

**Affiliations:** 1 Department of Visceral, Thoracic and Vascular Surgery, Faculty of Medicine and University Hospital Carl Gustav Carus, Technische Universität Dresden, Dresden, Germany; 2 National Center for Tumor Diseases (NCT), Dresden, Germany: German Cancer Research Center (DKFZ), Heidelberg, Germany, Faculty of Medicine and University Hospital Carl Gustav Carus, Technische Universität Dresden, Dresden, Germany; Helmholtz-Zentrum Dresden-Rossendorf (HZDR), Dresden, Germany; 3 German Cancer Consortium (DKTK), Partner Site Dresden, German Cancer Research Center (DKFZ), Heidelberg, Germany; 4 Institute of Clinical Chemistry and Laboratory Medicine, University Hospital Carl Gustav Carus, Technische Universität Dresden, Dresden, Germany; 5 Institute for Medical Informatics and Biometry, Faculty of Medicine Carl Gustav Carus, Technische Universität Dresden, Dresden, Germany; 6 Core Unit for Data Management and Analytics (CDMA), National Center for Tumor Diseases (NCT), Dresden, Germany; 7 Department of Surgery, University Medical Center Göttingen, Göttingen, Germany; 8 Institute for Transfusion Medicine, German Red Cross Blood Donation Service North-East, Dresden, Germany; 9 Experimental Transfusion Medicine, Faculty of Medicine Carl Gustav Carus, Technische Universität Dresden, Dresden, Germany; 10 Else Kröner Clinician Scientist Professor for Translational Tumor Immunological Research, 01307 Dresden, Germany

**Keywords:** Diagnostic markers, Pancreatic cancer, Prognostic markers

## Abstract

Pancreatic ductal adenocarcinoma (PDAC) responds poorly to systemic treatment, including new immunotherapeutic approaches. Biomarkers are urgently needed for early disease detection, patient stratification for treatment, and response prediction. The role of soluble CD40 (sCD40) is unknown in PDAC. In this study, we performed a quantitative multiplex analysis of 17 immune checkpoint proteins in serum samples from patients with various stages of PDAC in a discovery study (*n* = 107) and analyzed sCD40 by ELISA in a validation study (*n* = 317). Youden’s J statistic was used for diagnostic cut-off optimization. A Cox proportional hazards regression model was applied in an empiric approach for prognostic threshold optimization. Kaplan–Meier estimator and multivariable Cox regression analyses were used for survival analysis. sCD40 was significantly increased in the serum of patients with PDAC compared to healthy cohorts and patients with IPMN. In the validation cohort, the area under the receiver operating characteristic (ROC) c-statistic was 0.8, and combining sCD40 with CA19-9 yielded a c-statistic of 0.95. sCD40 levels were independent of the tumor stage. However, patients who received neoadjuvant chemotherapy had significantly lower sCD40 levels than those who underwent upfront surgery. Patients with a sCD40 level above the empirical threshold of 0.83 ng/ml had a significantly reduced overall survival with a hazard ratio of 1.4. This observation was pronounced in patients after neoadjuvant chemotherapy. Collectively, soluble CD40 may be considered as both a diagnostic and prognostic non-invasive biomarker in PDAC.

## Introduction

While some progress in the treatment of pancreatic ductal adenocarcinoma (PDAC) has been made, it still has a dismal prognosis with a 5-year overall survival rate of only 11%. PDAC is expected to become the second leading cause of cancer-related deaths in the United States by 2030^1,2^. New treatment approaches are urgently needed. After the first approval of the immune checkpoint inhibitor Ipilimumab (anti-CTLA-4) in 2011, several other immune modulators were developed, profoundly improving cancer treatment^3,4^. However, in PDAC, the use of immunotherapy has failed to show substantial improvements except for tumors with high mutational burden, as found in microsatellite instability (MSI) and mismatch repair deficiency (dMMR) that account for less than 1% of all PDACs^5–8^. PDAC is characterized by a low mutational burden and an immunosuppressive immune infiltrate associated with reduced survival^9^. However, recent analyses of human PDAC revealed that 20–30% of the patients display moderate T cell infiltration^10,11^. The lack of dendritic cell (DC)-mediated priming of T cells may be a possible mechanism for insufficient anti-tumor T cell immunity. Further, the activation of DCs is proposed as a putative strategy to push the immune system from an immunosuppressive myeloid state towards an anti-tumoral T cell response^12,13^. Agonistic CD40 antibody combined with gemcitabine/nab-paclitaxel treatment changed the immunosuppressive towards a T cell-dependent tumor rejecting microenvironment in a PDAC mouse model^14^. Besides T cell-related effects, agonistic CD40 treatment polarized tumor-infiltrating myeloid cells toward an anti-fibrotic phenotype and induced macrophages, depleting fibrosis and sensitizing tumors to chemotherapy^15,16^. Collectively, different mechanisms are at play in agonistic CD40 therapy. Preclinical and first clinical trials in various tumors, including results from phase 1 trials in PDAC, showed promising results^17–19^. The most recent results from the phase 2 PRINCE trial demonstrated an improved 1-year-survival in patients receiving nivolumab (anti-PD-1) and chemotherapy compared to the historical chemotherapy alone cohort, whereas the combined treatment with sotigalimab (agonistic anti-CD40) and chemotherapy displayed only a modestly improved 1-year-survival^20^. Thus, further investigations of CD40 in PDAC patients are warranted. Until now, data regarding the role of the soluble form of immune checkpoint proteins, including soluble CD40 (sCD40), are lacking. In this study, we investigated 17 soluble immune checkpoint proteins and identified sCD40 as a new diagnostic and prognostic biomarker in PDAC.

## Results

### sCD40 and sTIM-3 levels are significantly increased in PDAC patients compared to healthy controls

**Fig. 1:**
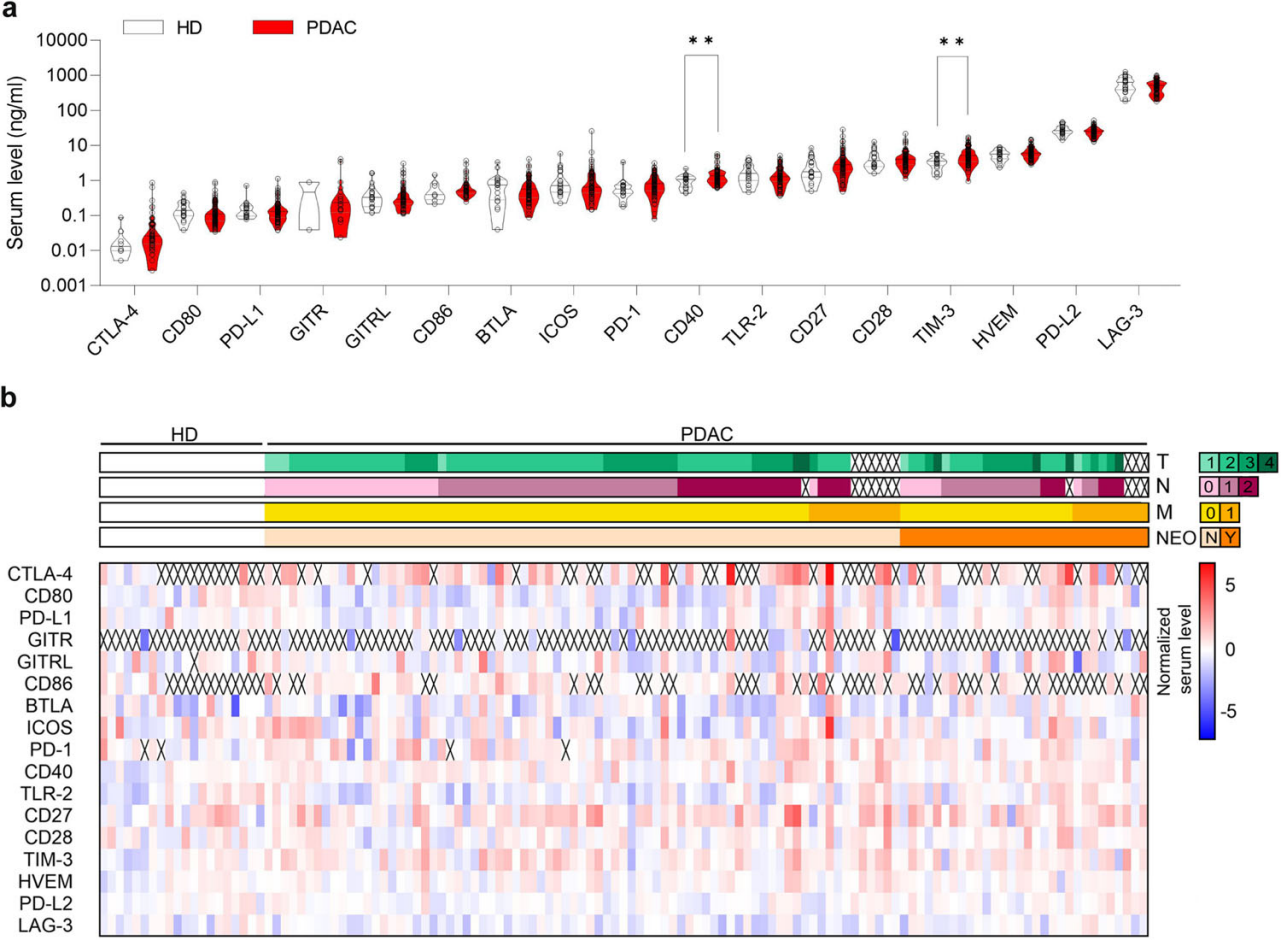
Serum level of 17 different immune checkpoint proteins measured in a discovery cohort of primarily resected PDAC patients (*n* = 77) and healthy donors (HD, *n* = 20). **a** Serum levels displayed as truncated violine plot with log10 scale. sTIM-3 and sCD40 with significantly higher levels in PDAC compared to HD (Mann Whitney test, sTIM-3 *P* = 0.003, sCD40 *P* = 0.007). **b** Unsupervised hierarchical clustering heatmap of serum levels; log2 normalized to the median of healthy donors. Measurements below the minimal detection concentrations are marked with crosses.

To screen for soluble immune checkpoints with clinical relevance, serum levels of 17 different proteins were determined in 107 PDAC patients and compared to samples from 20 healthy donors (HD). Luminex xMAP (multi-analyte profiling) technology, a bead-based multiplexed immunoassay, was used for analysis. Serum levels of the different immune checkpoints from PDAC patients with and without neoadjuvant therapy and controls are shown in Supplementary Fig. S1a and Fig. 1a and logarithm base 2 of the serum level relative to the median of healthy donors is presented as a heatmap (Fig. 1b). Soluble T cell immunoglobulin and mucin-domain containing-3 (sTIM-3) and soluble CD40 (sCD40) showed a significantly higher serum level in PDAC patients compared to controls in both cohorts. *CD40* mRNA expression ranked second highest in PDAC tumors among the 17 proteins analyzed with the TCGA data set. Comparing the mRNA level of *CD40* in PDAC and controls confirmed a significantly higher level in tumor tissue (Supplementary Fig. S1b, c).

### sCD40 is a diagnostic biomarker in human PDAC

**Fig. 2:**
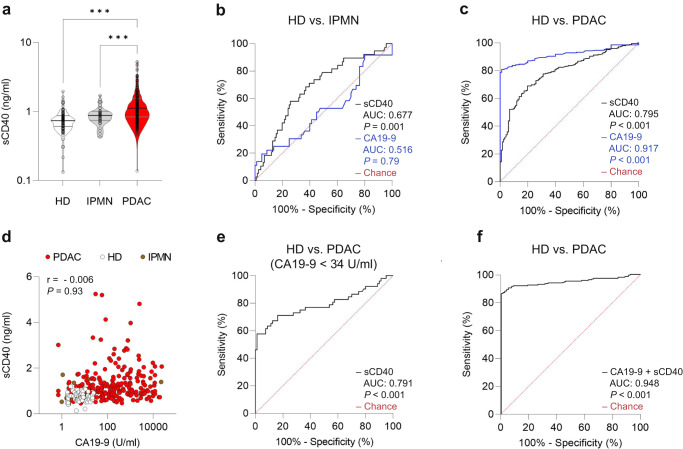
Diagnostic value of sCD40 in primarily resected PDAC patients. **a** Serum level of sCD40 from PDAC patients and healthy donors (one-way ANOVA using Tukey’s multiple comparisons test, HD vs. IPMN *P* = 0.48, IPMN vs. PDAC *P* < 0.001, HD vs. PDAC *P* < 0.001). **b** ROC of sCD40 and CA19-9 with an AUC significantly different to chance for sCD40 (*P* = 0.001, CI = 0.58–0.774, IPMN *n* = 38, healthy donors *n* = 116) and no significant differences to chance for CA19-9 (*P* = 0.79, CI 0.396–0.635, IPMN *n* = 38, healthy donors *n* = 80). **c** ROC of sCD40 and CA19-9 with an AUC significantly different to chance for sCD40 (*P* < 0.001, CI = 0.748–0.841, PDAC *n* = 251, healthy donors *n* = 116) and CA19-9 (*P* < 0.001, CI 0.885–0.948, PDAC *n* = 209, healthy donors *n* = 80). **d** Scatterplot with Pearson correlation of CA19-9 and sCD40 serum levels from PDAC patients and healthy donors (Pearson correlation only applied for PDAC samples, PDAC *n* = 209, HD *n* = 80). **e** ROC of sCD40 subcohort with patients showing CA19-9 level below the threshold of 34 U/ml. AUC is significantly different to chance (*P* < 0.001, 0.701–0.881, PDAC *n* = 52, healthy donors *n* = 80). **f** ROC of sCD40 and CA19-9 combined based on logistic regression analysis with an AUC significantly different to chance (*P* < 0.001, CI 0.923–0.974, PDAC *n* = 209, healthy donors *n* = 80).

A bicentric cohort of 317 PDAC patients was used for validation and further investigation. sCD40 serum levels were determined using ELISA. The sCD40 levels of PDAC patients were compared to healthy donors, confirming the significantly higher levels of sCD40 in PDAC patients without neoadjuvant pretreatment (primarily resected; PR). Additionally, the sCD40 serum level in PDAC patients was compared to that in IPMN patients. Significantly higher levels were found in PDAC, while IPMN and healthy donors did not differ significantly (Fig. 2a). Further, the sCD40 levels of PR patients were compared to the tumor marker CA19-9 as a binary classifier and plotted as receiver operating characteristic curve (ROC). Only moderate discrimination was found between healthy donors and IPMN patients (sCD40 AUC: 0.677 and CA19-9 AUC: 0.516) and between IPMN and PDAC patients (sCD40 AUC: 0.687 and CA19-9 AUC: 0.871; Fig. 2b and Supplementary Fig. S2a). While sCD40 reached an area under the curve (AUC) of 0.795, discriminating PDAC patients from healthy donors compared to CA19-9 with an AUC of 0.917 (Fig. 2c). An optimized sCD40 cut-off determined by Youden’s J statistic was found at a value of 0.913 ng/ml. No significant correlation between sCD40 and CA19-9 in PDAC was detected (Fig. 2d). Therefore, the combinational potential of both proteins was investigated. First, sCD40 was used as a binary classifier for the subpopulation of PDAC patients showing a CA19-9 level below the internal clinical threshold level of 34 U/ml based on the conservative translation of the German pancreatic cancer guideline. CA19-9 independent diagnostic capabilities of sCD40 were discovered (AUC: 0.791; Fig. 2e). Next, CA19-9 and sCD40 were used as a combined classifier applying logistic regression, further improving the ability of each single protein to discriminate healthy donors from PDAC patients (AUC: 0.948), while no beneficial discrimination of healthy donors from IPMN, or IPMN from PDAC patients were found (HD vs. IPMN AUC: 0.726; IPMN vs. PDAC AUC: 0.732; Fig. 2f and Supplementary Fig. S2b, c). For further visualization of sCD40 diagnostic accuracy in patients without neoadjuvant treatment, see the contingency table (Supplementary Table S9).

### High sCD40 levels are associated with reduced overall survival

**Fig. 3:**
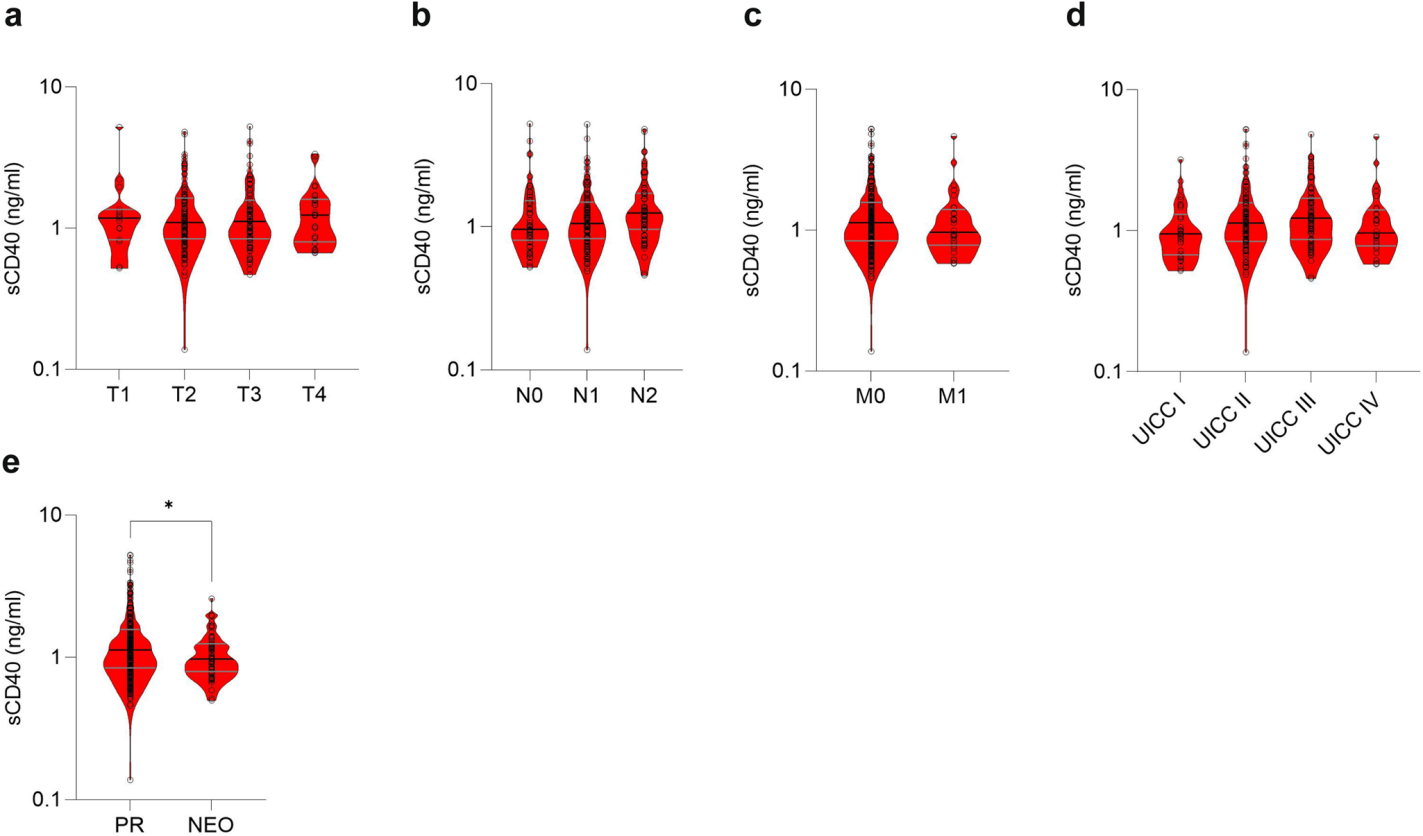
Correlation between sCD40 and tumor stage or treatment in PDAC patients. **a** Serum level of sCD40 in different T stages. No significant differences could be measured (one-way ANOVA using Tukey’s multiple comparisons test, T1 vs. T2 *P* = 0.982, T1 vs. T3 *P* = 0.986, T1 vs. T4 *P* = 0.99, T2 vs. T3 *P* > 0.999, T2 vs. T4 *P* = 0.999, T3 vs. T4 *P* = 0.999). **b** Serum level of sCD40 in different N stages. No significant differences could be measured (one-way ANOVA using Tukey’s multiple comparisons test, N0 vs. N1 *P* = 0.958, N0 vs. N2 *P* = 0.337, N1 vs. N2 *P* = 0.133). **c** Serum levels of sCD40 compared between patients with M0 and M1 stage. No significant differences could be measured (two-tailed unpaired *t* test, M0 vs. M1 *P* = 0.623). **d** Serum levels of sCD40 in different UICC stages. No significant differences could be measured (one-way ANOVA using Tukey’s multiple comparisons test, UICC I vs. UICC II *P* = 0.323, UICC I vs. UICC III *P* = 0.185, UICC I vs. UICC IV *P* = 0.847, UICC II vs. UICC III *P* = 0.955, UICC II vs. UICC IV *P* = 0.931, UICC III vs. UICC IV *P* = 0.789). **e** Serum level of sCD40 compared between patients treated with primary resection (PR) or neoadjuvant chemotherapy (NEO) followed by surgery. Significant differences could be measured (two-tailed unpaired *t* test, PR vs. NEO *P* = 0.012).

**Fig. 4:**
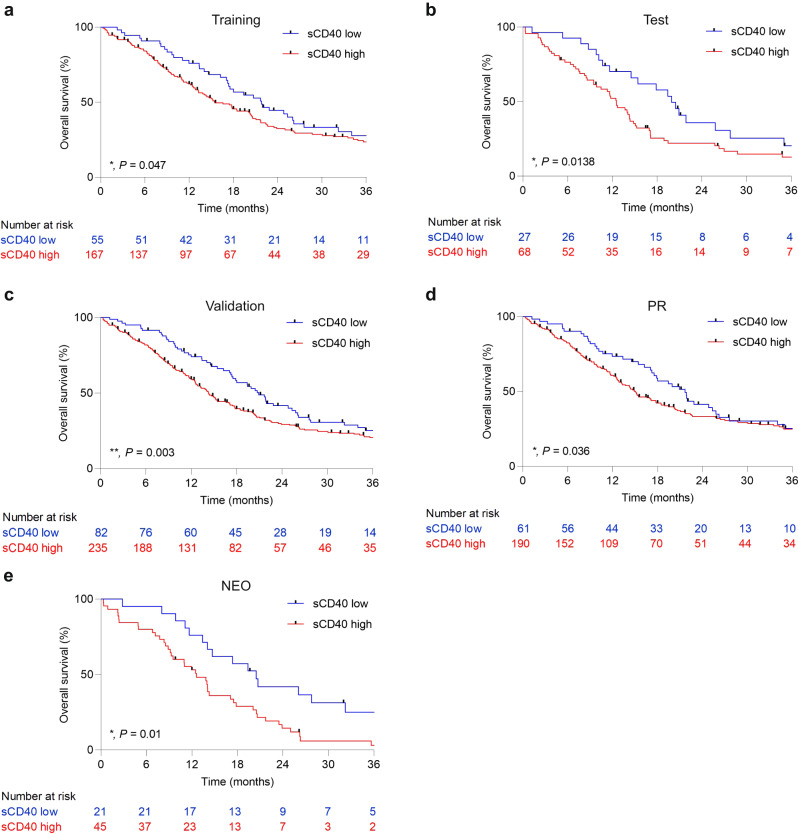
sCD40 as a prognostic marker. **a** Kaplan–Meier curve of patients with high or low sCD40 levels in the training cohort. Threshold of 0.835 ng/ml was applied. (Median survival of sCD40 low vs. high: 21.83 vs. 15.47 months, Log-rank *P* = 0.153, Gehan–Breslow–Wilcoxon *P* = 0.047). **b** Kaplan–Meier curve of patients with high or low sCD40 levels in the test cohort. Threshold of 0.835 ng/ml was applied. (Median survival of sCD40 low vs. high: 19.93 vs. 12.57 months, Log-rank *P* = 0.04, Gehan–Breslow–Wilcoxon *P* = 0.0138). **c** Kaplan–Meier curve of patients with high or low sCD40 levels in the complete validation cohort. Threshold of 0.835 ng/ml was applied. (Median survival of sCD40 low vs. high: 20.93 vs. 14.3 months, Log-rank *P* = 0.03, Gehan–Breslow–Wilcoxon *P* = 0.003). **d** Kaplan–Meier curve of patients with high or low sCD40 levels in the primary resected cohort. Threshold of 0.835 ng/ml was applied. (Median survival of sCD40 low vs. high: 21.67 vs. 14.97 months, Log-rank *P* = 0.183, Gehan–Breslow–Wilcoxon *P* = 0.036). **e** Kaplan–Meier curve of patients with high or low sCD40 levels in the neoadjuvant-treated cohort. Threshold of 0.835 ng/ml was applied. (Median survival of sCD40 low vs. high: 20.47 vs. 12.57 months, Log-rank *P* = 0.006, Gehan–Breslow–Wilcoxon *P* = 0.01).

Next, we investigated the association of sCD40 with tumor stage in primary resected or neoadjuvant-treated patients. No significant difference in sCD40 concentrations between each T, N, M and UICC stage was detected. However, patients who underwent neoadjuvant chemotherapy (NEO) before surgery revealed a significantly lower level of sCD40 compared to patients who were primarily resected (PR; Fig. 3a–e and Supplementary Fig. S3a–d). To further investigate the prognostic role of sCD40, the impact of high and low sCD40 serum levels on overall survival was analyzed. An empirical approach with Cox regression was used for threshold determination. Following the hold-out method, a randomized training cohort of 70% and a test cohort of 30% of the complete validation cohort were built (Supplementary Tables S3–S5). This led to the most distinct hazard ratio for patients with sCD40 > 0.835 ng/ml in the training cohort and was validated by applying this threshold to the test cohort. The Kaplan–Meier survival curve showed a significantly reduced overall survival for patients with high sCD40 level in training-, test- and when applied on the complete validation cohort (Fig. 4a–c and Table 1). A multivariable Cox regression analysis confirmed a significantly higher hazard ratio for patients with high sCD40 level (Table 2). Survival analysis on neoadjuvant-treated patients revealed a pronounced association between sCD40 and overall survival (Fig. 4d, e). Separating the cohort according to the patients’ tumor stage revealed the most prominent association of sCD40 on overall survival in patients with metastatic PDAC (UICC IV; Supplementary Fig. S4a–d). Significantly worse disease-free survival (DFS) was detected in the sCD40 high patients with UICC IV stage, the complete validation, and the neoadjuvant-treated cohort (Supplementary Fig. S4a–g). For comparison, we also analyzed the prognostic value of CA19-9. The same empirical approach was used, giving a threshold of 85 U/ml, uncovering a worse overall survival for high CA19-9 levels while no significant differences in disease-free survival were observed (Supplementary Fig. S5a). Separating the groups into PR and NEO cohorts revealed only in PR patients a significantly superior overall and disease-free survival in patients with low CA19-9 levels (Supplementary Fig. S5b, c). Combining both markers increased the prognostic capabilities compared to sCD40 alone. The hazard ratio for PDAC patients with CA19-9 and sCD40 levels above thresholds ( > 85 U/ml and >0.835 ng/ml) compared to patients below those thresholds improved from 1.393 to 1.908 (Table 1). In Kaplan–Meier analyses, the combination of both markers presented a significantly reduced outcome with significantly worse overall and disease-free survival for the CA19-9 + sCD40 high cohort (Supplementary Fig. S5d). Further, high mRNA levels of *CD40* in tumor samples were associated with worse overall survival, whereas no significant difference in DFS was observed (Supplementary Fig. S1d, e). Notably, age was significantly different between the sCD40 high and low groups, but no direct correlation was detected, and the standardized mean difference (SMD) between patients with high and low sCD40 level was low (SMD = 0.311; Supplementary Fig. S6a). Together, this indicates that age is not a significant confounder in this study.

**Table 1:** Univariate Cox regression analysis.

	HR (95% CI)	*P* value	*n*
sCD40 low			
high	1.393 (1.026–1.892)	0.034	317
sCD40 + CA19 low			
high	1.908 (1.114–3.266)	0.019	145

**Table 2:** Multivariate Cox regression analysis (*n* = 209).

	HR (95% CI)	*P* value
sCD40 low		
high	1.563 (1.002–2.438)	0.049
T1		
2	1.258 (0.605–2.618)	0.539
3	2.293 (1.08–4.869)	0.031
4	2.262 (0.477–10.733)	0.304
N0		
1	2.015 (1.052–3.861)	0.035
2	1.727 (0.502–5.946)	0.386
M0		
1	0.549 (0.191–1.581)	0.267
NEO No		
Yes	1.859 (0.959–3.602)	0.066
R0		
1	1.399 (0.873–2.24)	0.163
2	0.603 (0.051–7.058)	0.687
x	1.998 (0.365–10.94)	0.425
G0		
1	0.727 (0.037–14.236)	0.834
2	1.022 (0.112–9.343)	0.985
3	1.45 (0.157–13.373)	0.743
x	1.096 (0.107–11.275)	0.938
UICC I		
II	0.538 (0.229–1.266)	0.156
III	0.768 (0.196–3.006)	0.704
IV	NA	NA
Age		
	1.013 (0.993–1.032)	0.2
CA19-9		
	0.976 (0.659–1.444)	0.902

## Discussion

The dismal prognosis of PDAC is closely related to its late diagnosis. At the time of diagnosis, only 15–20% of the patients present with a resectable tumor. The lack of specific symptoms impedes early detection^1,21,22^. Accordingly, diagnostic biomarkers are needed. Currently, CA19-9 is the most reliable serum biomarker, with a sensitivity of 79–81% and a specificity of 82–90% in symptomatic patients^23^. It is a good marker for therapy monitoring, but falls short as a screening marker^24,25^. Moreover, some patients do not present with elevated CA19-9 at all. Several different biomarkers were studied but have yet to be established in clinical routine^26^. Our study showed that sCD40 may be an additional diagnostic marker. Combining a possible marker with CA19-9 further increased the accuracy of diagnosis in different studies^27,28^. sCD40 combined with CA19-9 also increased its diagnostic value in PDAC patients. However, in our study, we observed a diagnostic accuracy above average for CA19-9, which needs to be considered in future applications^29^. Surgical resection is the only possible curative treatment, with a 5-year survival rate of only 27% and 5-year recurrence-free survival of only 11%, indicating the need for further systemic treatment strategies^30^. Until now, immunotherapy in pancreatic cancer failed its expectations, and only patients with mismatch repair deficiency and high mutational burden showed to benefit from this treatment strategy. Further approaches for patient stratification are warranted. Several studies described promising methods for patient stratifications, but mainly focused on intratumoral factors^8,31–33^. Little is known about the role of soluble immune checkpoint proteins. Different studies have been pointing toward a significant relevance of soluble immune checkpoints for immunotherapy, either through direct interaction with their corresponding receptor/ligand on cells or through blockade with immunomodulating drugs. Overall, soluble immune checkpoints may be used for response prediction and patient stratification^34–37^. Currently, only a few proteins, including PD-1, PD-L1, pan-BTN3As, BTN3A1, BTLA, and CD40L, have been evaluated in their soluble form in PDAC^37–40^. sCD40 has not been investigated in PDAC. In clear cell renal cell cancer, soluble TIM-3 was associated with advanced disease stage and reduced survival^41^. TIM-3 binds to galectin-9, which is elevated in PDAC patients^42,43^. This highlights a potential diagnostic value of sTIM-3, which we also found to be increased in the serum of PDAC patients. sCD40 was elevated and associated with poor prognosis in multiple myeloma and acute myeloid leukemia^44,45^. Our results uncovered a significant prognostic role of sCD40 in PDAC. Strikingly, the most pronounced effect on prognosis was found in neoadjuvant-treated patients, while CA19-9 was not a prognostic marker in this group. Previous findings of altered immune cell infiltration after neoadjuvant treatment point to an immune-associated mechanism^46,47^. Considering the preliminary results from the current NORPACT-1 study, markers like sCD40 for possible treatment stratification before neoadjuvant therapy are warranted^48^. Recently, results from phase 1 and 2 trials using agonistic CD40 antibody (sotigalimab) in metastatic PDAC were published^19,20^. While preclinical studies showed promising results, the phase 2 clinical trial data failed to fulfill its primary endpoint. However, sotigalimab combined with chemotherapy showed a trend towards an increased 1-year overall survival compared to a historical control with chemotherapy only^20,49^. Previously, systemic inflammation markers like C-reactive protein (CRP), serum amyloid A (SAA), and neutrophil/lymphocyte ratio (NLR) were found to correlate with poor survival in PDAC^50–54^. Moreover, systemic inflammation may be linked to the therapy outcome of immunotherapy in PDAC^55–57^. CD40 is known to play an essential role in the inflammation pathway, which may link our results to the previously described relevance of systemic inflammation for the outcome of PDAC patients. Notably, systemic inflammation is also described as a putative mechanism for treatment failure of agonistic CD40-based therapies^58^. Thus, CD40 and related pathway proteins should be considered in future CD40-based immunotherapeutic trials. Even in the case of the promising multi-specific anti-CD40 DARPin construct, activated by fibroblast activation protein (FAP) at the tumor site, an interaction of soluble CD40 may occur and should be considered for future studies^59^.

Collectively, our study highlights the relevance of soluble immune checkpoints in cancer. sCD40 may be used as an additional diagnostic non-invasive serum marker for differential diagnosis and early detection of PDAC. Furthermore, it is a prognostic marker. It may be used for patient stratification, mainly in neoadjuvant-treated patients, while it also should be considered as a biomarker for future CD40-based immunotherapy studies.

## Methods

### Patient samples

Serum samples from 107 patients with PDAC who underwent surgical resection at the Department of Visceral, Thoracic and Vascular Surgery at the University Hospital Carl Gustav Carus Dresden (Dresden, Germany) were collected between 2005 and 2019 and used as a discovery cohort. Serum samples from partly additional 317 PDAC patients from Dresden (*n* = 181) and the Clinic of General, Visceral and Pediatric Surgery, University Medical Center Göttingen (Göttingen, Germany, *n* = 136) between 2006 and 2019 were used as a validation cohort. Samples were chosen from the biobank sequentially. All PDACs were histologically confirmed. Venous blood was drawn into serum separator tubes on the day of surgery or up to 10 days before surgery. All samples were centrifuged immediately (30 min–4 h after collection, 12 min, 1500 × *g*, 4 °C), and the aliquoted serum was stored at -80 °C without delay after centrifugation. Only aliquots with maximal four freeze-thaw cycles were used. Serum from healthy donors (*n* = 20, screening cohort; *n* = 116, validation cohort) was obtained at the Department of Visceral, Thoracic and Vascular Surgery at the University Hospital Carl Gustav Carus (Dresden, Germany) or the German Red Cross blood donation in the manner mentioned above. A person was considered a healthy donor when no present or past tumor disease or active disease with an immune response was known (52% blood donors, 27% varicosis patients, 15% hernia patients, 6% others (lipoma, vascular malformation). All patients and healthy donors gave written informed consent, and the study was approved by the Ethics Committee of the TU Dresden (Ref-Nr.: 446112017) or the Ethics Committee Göttingen (Ref-Nr.: 24/4/03), respectively. Clinical tumor stages were determined according to the TNM classification system (UICC; Edition 8, stages were updated according to the pathological information if needed). Clinical characteristics are shown in Table 3 and Supplementary Tables S1–8. The whole study was performed following the STARD and REMARK protocol (Supplementary Fig. S6b)^60,61^.

**Table 3:** Clinicopathologic characteristics of patients in validation cohort.

	sCD40 low *n* = 82		sCD40 high *n* = 235		
	*n*	%	*n*	%	*P* value
Age					
Median (range)	66.50	(28–88)	69.00	(31–89)	0.015^a^
Gender					
Female	45	54.88	116	49.36	0.39^b^
Male	37	45.12	119	50.64	
pT stage					
1	6	7.32	14	5.96	0.946^b^
2	31	37.80	97	41.28	
3	26	31.71	74	31.49	
4	11	13.41	30	12.77	
Unknown	8	9.76	20	8.51	
pN stage					
0	25	30.49	51	21.70	0.078^b^
1	38	46.34	102	43.40	
2	13	15.85	63	26.81	
Unknown	6	7.32	19	8.09	
cM stage					
0	70	85.37	207	88.09	0.406^b^
1	12	14.63	28	11.91	
UICC stage					
I	14	17.07	28	11.91	0.437^b^
II	34	41.46	104	44.26	
III	20	24.39	74	31.49	
IV	12	14.63	28	11.91	
Unknown	2	2.44	0	0.00	
Neoadjuvant treatment					
Yes	21	25.61	45	19.15	0.153^b^
No	54	65.85	179	76.17	
Unknown	7	8.54	11	4.68	

^a^*t* test.

^b^Chi-squared test.

### TCGA and GTEx RNA-Seq analysis

mRNA amounts were assessed in The Cancer Genome Atlas (TCGA) RNA-seq data sets using the cBioPortal for Cancer Genomics. mRNA values in fragments per kilobase million (FPKM) were used, and clinical correlations were performed using GraphPad Prism 9.0 (GraphPad Software, La Jolla, CA). GEPIA, a web server for cancer and normal gene expression profiling and interactive analyses, was used to compare gene expression between PDAC and healthy controls and for survival analyses. All data was based on the TCGA and the Genotype-Tissue Expression (GTEx) project^62^.

### Quantification of soluble checkpoints

Seventeen different soluble immune checkpoint proteins (BTLA, CD27, CD28, TIM-3, HVEM, CD40, GITR, LAG-3, TLR-2, GITRL, PD-1, CTLA-4, CD80, CD86, PD-L1, PD-L2, ICOS) were measured using the bead-based Luminex® Multi-Analyte Profiling (xMAP) technology according to the manufacturer’s instruction (MILLIPLEX, catalog #: HCKP1-11K, EMD Millipore Corporation, Billerica, USA). Briefly, 25 µL of 1:2 diluted serum samples were mixed with fluorescent-coded magnetic beads coated with analyte-specific capture antibodies and incubated overnight. Subsequently, a mixture of biotinylated detection antibodies specific to the seventeen analytes was added, followed by Streptavidin-Phycoerythrin. The median fluorescence intensity (MFI) was measured by a Luminex200 machine. The MFI was converted to the protein concentration using a standard curve fitted with a 5-parameter logistic model by the xPONENT software. Quality control was run and counter-checked according to the manufacturer’s protocol. The minimal detection concentration (ng/mL) for each protein was: BTLA 0.0259, CD27 0.0165, CD28 0.0655, TIM-3 0.0012, HVEM 0.0006, CD40 0.0027, GITR 0.0064, LAG-3 0.0372, TLR-2 18, GITRL 0.0173, PD-1 0.008, CTLA-4 0.0058, CD80 0.0093, CD86 0.0575, PD-L1 0.0011, PD-L2 0.0453, ICOS 0.0394. Enzyme-linked immunosorbent assay (ELISA) was used according to the manufacturer’s protocol for the detection of soluble CD40 (Human CD40 Quantikine ELISA Kit, Catalog #: DCCD40, R&D Systems, Minneapolis, USA). The assay’s sensitivity was 0.00163 ng/mL. Varioskan LUX (Thermo Fischer, Waltham, USA) was used for detection. An average intra-variability of 1.962% with a SD of 5.347% and an average inter-variability of 13.813% with a SD of 12.86% occurred (Bland-Altman statistics). All measurements were performed at the laboratory of the Department of Surgery at the University Hospital Carl Gustav Carus Dresden. CA19-9 values were retrieved from the clinical laboratories. Analyses were conducted by the certified Institute of Laboratory Medicine at the University Hospital Carl Gustav Carus Dresden or University Hospital Göttingen, respectively.

### Statistical analysis

Data is presented in violin plots with median and quartiles, scatter, or box plots. An unpaired, two-tailed Student’s *t* test was used for the comparison of two groups. One-way ANOVA with Tukey statistics was used for groups of more than two. For cut-off optimization, Youden’s J statistic, in combination with receiver operating characteristic (ROC), was applied. Kaplan–Meier with Gehan–Breslow–Wilcoxon test was used for survival analysis. For threshold optimization of overall survival, the results from Youden’s J statistic were used as starting point and empirically improved in steps of ±0.5, 0.05, 0.005, and 0.001 ng/ml for sCD40 and in steps of ±10, 1, 0.5, 0.05, 0.01 U/ml for CA19-9. A *P* value of ≤0.05 was considered significant. A confidence interval of 95% was used when stated. Logistic and Cox regression were performed using R statistical software (Version 4.2.0. R Core Team, 2022). R: A language and environment for statistical computing. R Foundation for Statistical Computing, Vienna, Austria). GraphPad Prism 9.0 (GraphPad Software, La Jolla, USA) was used for all other analyses.

### Reporting summary

Further information on research design is available in the Nature Research Reporting Summary linked to this article.

### Supplementary information

Supplementary Information

REPORTING SUMMARY

## Data Availability

The data supporting this study’s findings are available from the corresponding author upon reasonable request.
