# Supplementary material for: Serum immune checkpoint profiling identifies soluble CD40 as a biomarker for pancreatic cancer
Source: NPJ Precis Oncol. 2023 Oct 14;7:104. doi: 10.1038/s41698-023-00459-9 (PMC10576756; doi:10.1038/s41698-023-00459-9)
Supplement: Supplementary file 1 — Supplementary Information [file 41698_2023_459_MOESM1_ESM.pdf]

Supplementary Figure S1

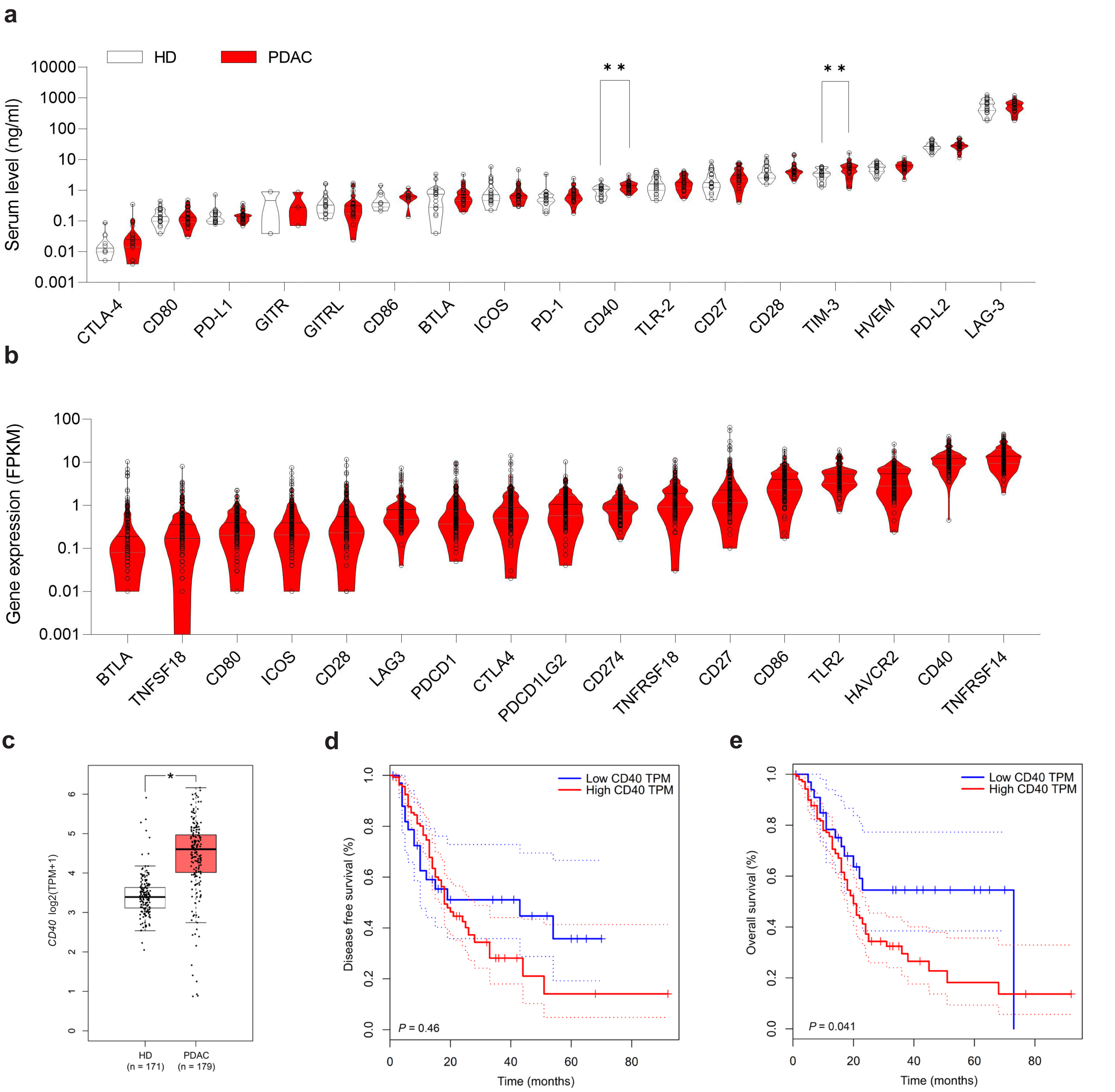

Supplementary Figure S1. Serum level and gene expression of 17 different immune checkpoint proteins measured in cancer tissue of PDAC patients and the role of CD40 as a diagnostic and prognostic marker. **a** Serum level of 17 different immune checkpoint protein measured in neoadjuvant treated discovery cohort of PDAC patients (n= 30) and healthy donors (HD, n = 20). Serum levels are displayed as truncated violine plots with log10 scale. sTIM-3 and sCD40 with significantly higher levels in PDAC compared to HD (Mann Whitney test, sTIM3 P = 0.005, sCD40 P = 0.006). **b** Gene expression levels displayed as truncated violine plot with log10 scale of fragments per kilo base of transcript per million mapped fragments (FPKM). The genes are ordered after their expression levels. Data was obtained from The Cancer Genome Atlas (TCGA, n = 146). **c** Gene expression of CD40 in PDAC compared to expression in healthy control organs. Displayed in log2 of transcripts per million (TPM+1). Data was obtained from TCGA and the GTEx project and processed with the GEPIA web service (PDAC n = 179, HD = 171). **d-e** Disease free and overall survival of CD40 low and high expressing patients (lower 20% and upper 80%). Data was obtained from TCGA and processed with GEPIA web service (low CD40 n = 36, high CD40 n = 142).

Supplementary Figure S2

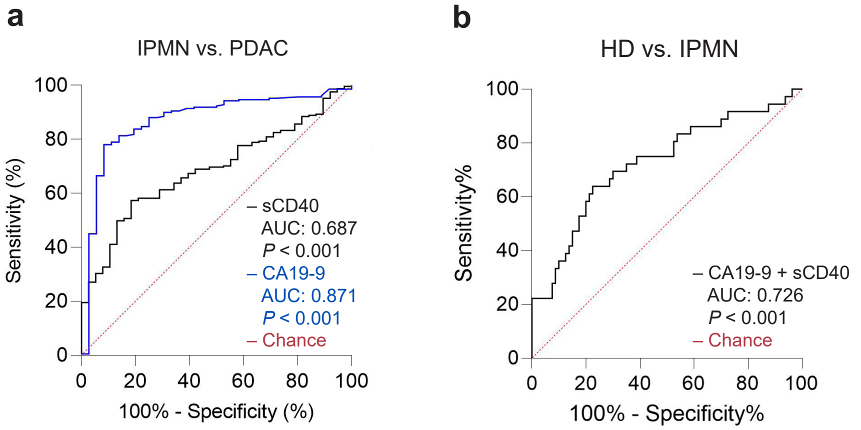

Supplementary Figure S2. Diagnostic value of sCD40 in primarily resected PDAC patients. **a** ROC of sCD40 and CA19-9 with an AUC significantly different to chance for sCD40 (P < 0.001, CI = 0.612 to 0.761, IPMN n = 38, PDAC n = 251) and for CA19-9 (P < 0.001, CI 0.806 to 0.936, IPMN n = 38, PDAC n = 209). **b** ROC of sCD40 and CA19-9 combined based on logistic regression analysis with an AUC significantly different to chance (P < 0.001, CI 0.621 to 0.83, HD n = 80, IPMN n = 38). **c** ROC of sCD40 and CA19-9 combined based on logistic regression analysis with an AUC significantly different to chance (P < 0.001, CI 0.653 - 0.811, IPMN n = 38, PDAC n = 209).

Supplementary Figure S3

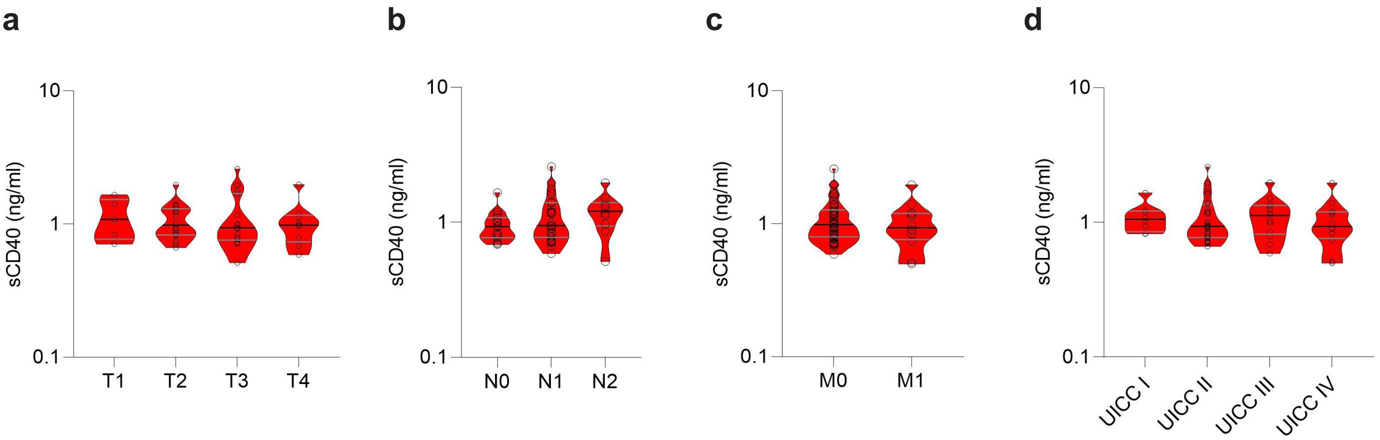

Supplementary Figure S3. Correlation between sCD40 and tumor stage in neoadjuvant treated PDAC patients. **a** Serum level of sCD40 in different T states. No significant differences could be measured (one-way Anova using Tukey‘s multiple comparisons test, T1 vs. T2 P = 0.995, T1 vs. T3 P = 0.999, T1 vs. T4 P = 0.978, T2 vs. T3 P > 0.956, T2 vs. T4 P = 0.994, T3 vs. T4 P = 0.913). **b** Serum level of sCD40 in different N state. No significant differences could be measured (one-way Anova using Tukey‘s multiple comparisons test, N0 vs. N1 P = 0.248, N0 vs. N2 P = 0.256, N1 vs. N2 P = 0.908). **c** Serum levels of sCD40 compared between patients with M0 and M1. No significant differences could be measured (two-tailed unpaired t test, M0 vs. M1 P = 0.507). **d** Serum levels of sCD40 in different UICC states. No significant differences could be measured (one-way Anova using Tukey‘s multiple comparisons test, UICC I vs. UICC II P = 0.999, UICC I vs. UICC III P = 0.999, UICC I vs. UICC IV P = 0.978, UICC II vs. UICC III P = 0.999, UICC II vs. UICC IV P = 0.904, UICC III vs. UICC IV P = 0.923).

Supplementary Figure S4

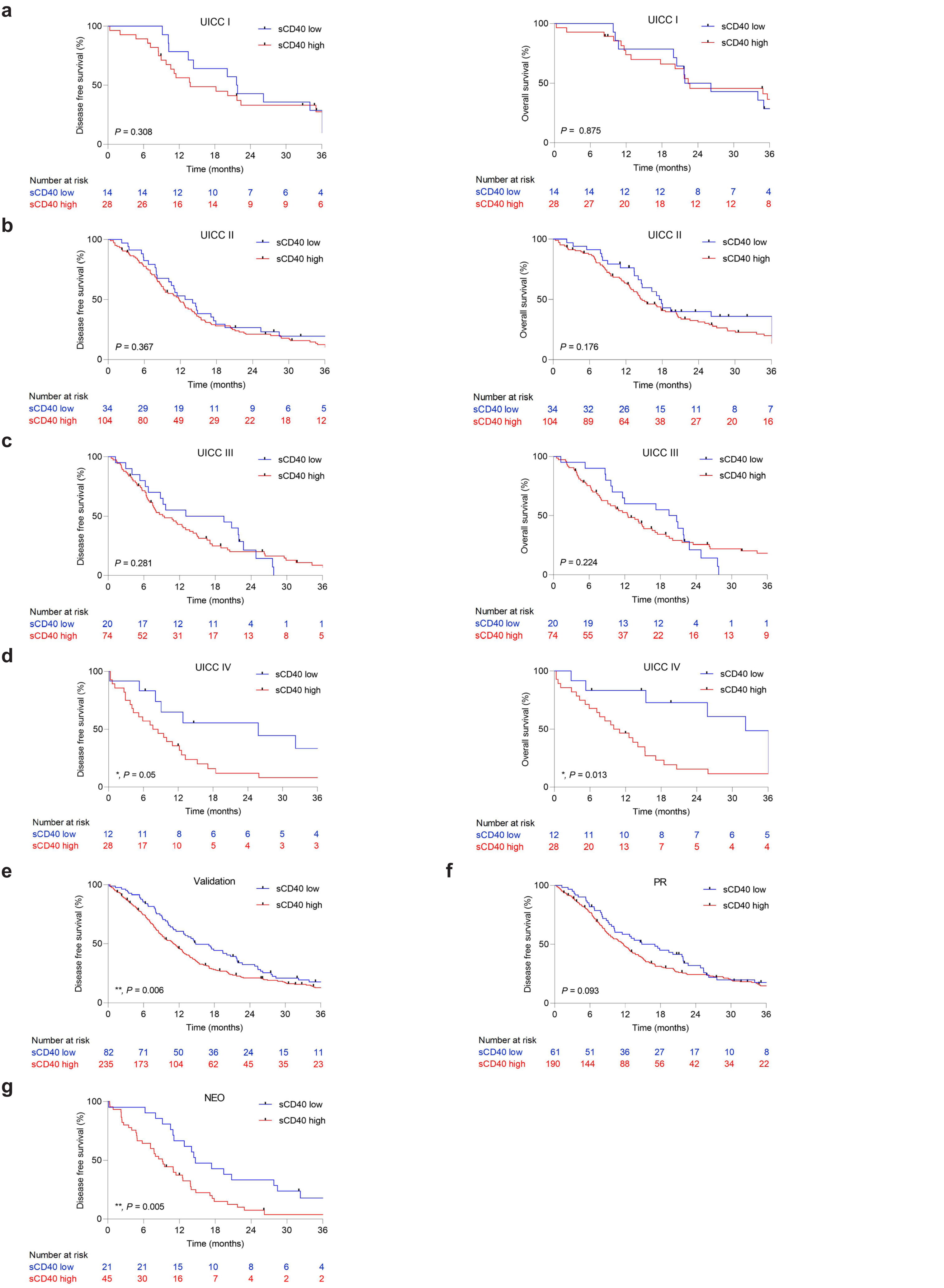

Supplementary Figure S4. sCD40 as a prognostic marker. **a-d** Kaplan-Meier curves of disease free (DFS) and overall survival (OS) of patients with high or low sCD40 levels in UICC I-IV cohorts. Threshold of 0.835 ng/ml was applied. (UICC I: DFS Log-rank P = 0.787, Gehan-Breslow-Wilcoxon P = 0.308; OS Log-rank P = 0.524, Gehan-Breslow-Wilcoxon P = 0.875; UICC II: DFS Log-rank P = 0.368, Gehan-Breslow-Wilcoxon P = 0.367; OS Log-rank P = 0.216, Gehan-Breslow-Wilcoxon P = 0.176; UICC III: DFS Log-rank P = 0.659, Gehan-Breslow-Wilcoxon P = 0.281; OS Log-rank P = 0.919, Gehan-Breslow-Wilcoxon P = 0.224; UICC IV: DFS Log-rank P = 0.032, Gehan-Breslow-Wilcoxon P = 0.05; OS Log-rank P = 0.006, Gehan-Breslow-Wilcoxon P = 0.013). **e** Kaplan-Meier curve for disease free survival of patients with high or low sCD40 levels in the complete validation cohort. Threshold of 0.835 ng/ml was applied. (Log-rank P = 0.03, Gehan-Breslow-Wilcoxon P = 0.006). **f-g** Kaplan-Meier curve for disease free survival of patients with high or low sCD40 levels in PR or NEO cohort. Threshold of 0.835 ng/ml was applied. (PR: Log-rank P = 0.234, Gehan-Breslow-Wilcoxon P = 0.093; NEO: Log-rank P = 0.004, Gehan-Breslow-Wilcoxon P = 0.005).

Supplementary Figure S5

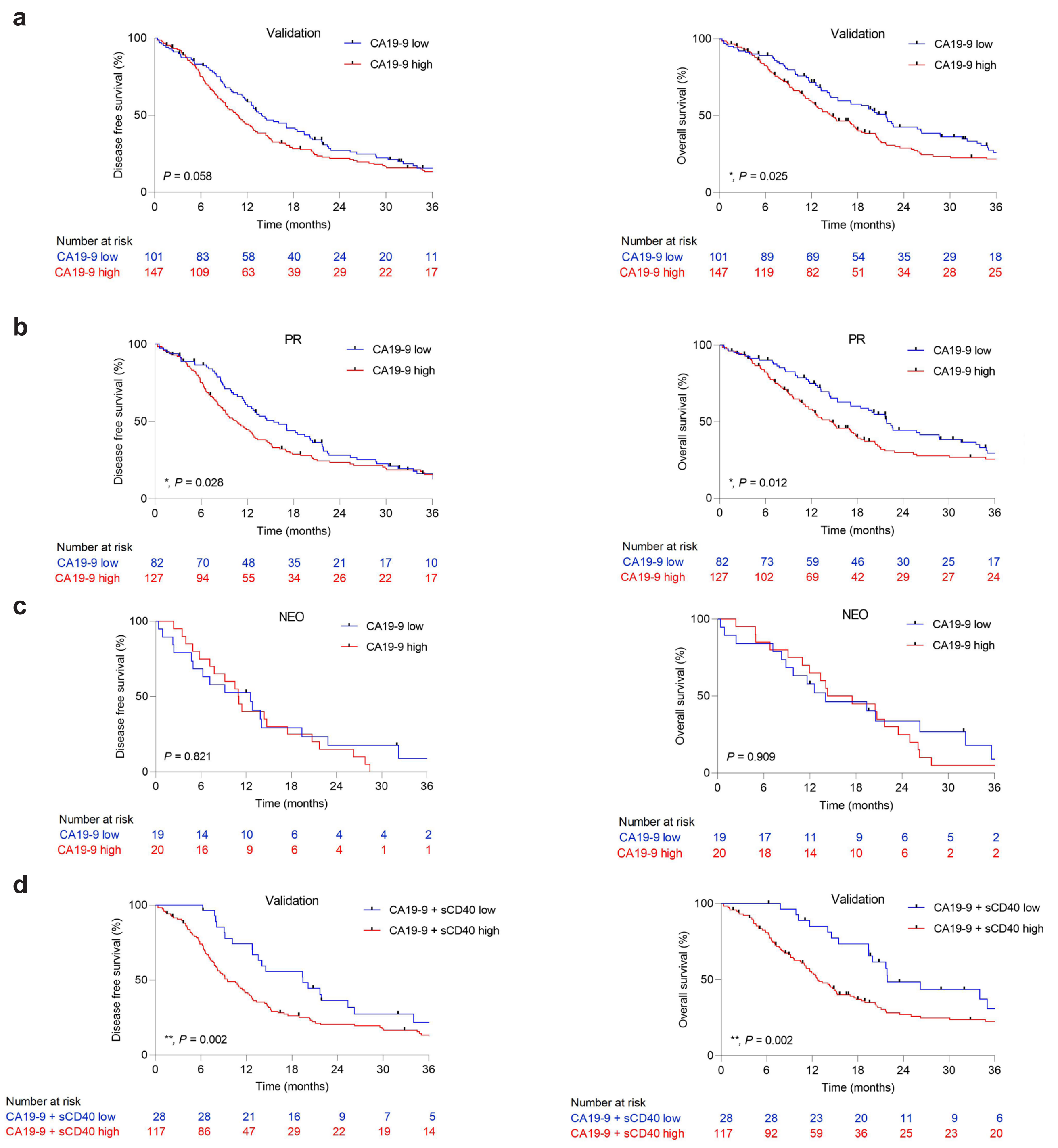

Supplementary Figure S5. CA19-9 as a prognostic marker. **a** Kaplan-Meier curves of disease free (DFS) and overall survival (OS) of patients with high or low CA19-9 levels in complete validation cohort. Threshold of 85 U/ml was applied. (DFS Log-rank P = 0.137, Gehan-Breslow-Wilcoxon P = 0.058; OS Log-rank P = 0.054, Gehan-Breslow-Wilcoxon P = 0.025). **b-c** Kaplan-Meier curves of DFS and OS of patients with high or low CA19-9 levels in PR and NEO cohort. Threshold of 85 U/ml was applied. (PR: DFS Log-rank P = 0.167, Gehan-Breslow-Wilcoxon P = 0.028; OS Log-rank P = 0.05, Gehan-Breslow-Wilcoxon P = 0.012; NEO: DFS Log-rank P = 0.546, Gehan-Breslow-Wilcoxon P = 0.821; OS Log-rank P = 0.58, Gehan-Breslow-Wilcoxon P = 0.909). **d** Kaplan-Meier curves of DFS and OS of patients with high or low CA19-9 and sCD40 levels in the complete validation cohort. Threshold of 85 U/ml and 0.835ng/ml was applied. (DFS Log-rank P = 0.019, Gehan-Breslow-Wilcoxon P = 0.002; OS Log-rank P = 0.017, Gehan-Breslow-Wilcoxon P = 0.002).

Supplementary Figure S6

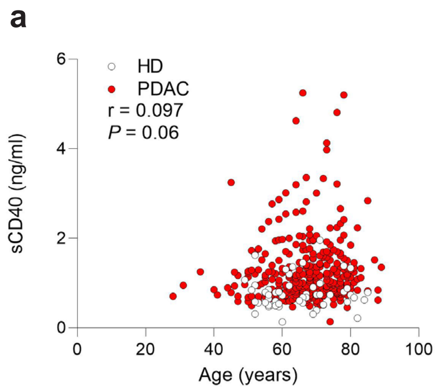

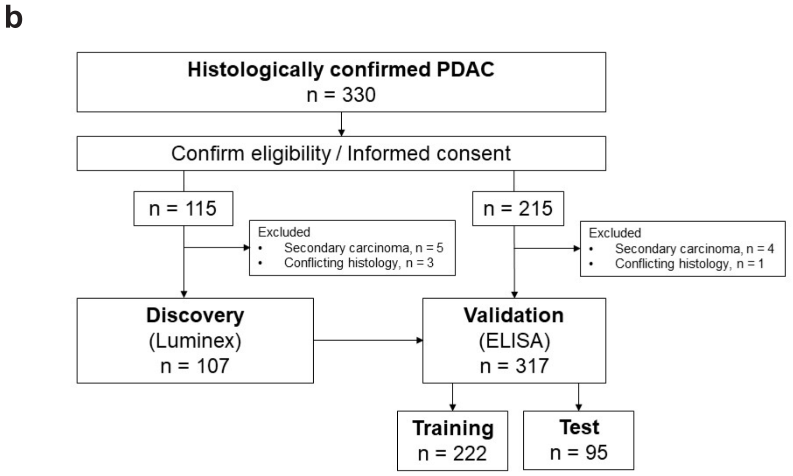

Supplementary Figure S6. a Scatter plot and Pearson correlation of sCD40 and age from validation cohort to rule out age as a confounder (n = 317). **b** Flow chart of patient selection and study procedure.

Supplementary Table S1.
Clinicopathologic characteristics - Discovery cohort

	Complete n = 107		PR n = 77		NEO n = 30		
	n	%	n	%	n	%	p-value
**Age**							
Median (range)	68.00	(28-83)	69.00	(40-83)	65.00	(28-79)	**0.008**[a]
**Gender**							
female	51	47.66	36	46.75	15	50.00	0.763[b]
male	56	52.34	41	53.25	15	50.00	
**pT Stage**							
1	7	6.54	4	5.19	3	10.00	0.054[b]
2	57	53.27	46	59.74	11	36.67	
3	28	26.17	19	24.68	9	30.00	
4	6	5.61	2	2.60	4	13.33	
Unknown	9	8.41	6	7.79	3	10.00	
**pN Stage**							
0	28	26.17	22	28.57	6	20.00	0.542[b]
1	43	40.19	29	37.66	14	46.67	
2	25	23.36	19	24.68	6	20.00	
Unknown	11	10.28	7	9.09	4	13.33	
**cM Stage**							
0	87	81.31	66	85.71	21	70.00	0.061[b]
1	20	18.69	11	14.29	9	30.00	
**UICC Stage**							
I	20	18.69	17	22.08	3	10.00	0.205[b]
II	45	42.06	33	42.86	12	40.00	
III	22	20.56	16	20.78	6	20.00	
IV	20	18.69	11	14.29	9	30.00	
**Neoadjuvant Treatment**							
Yes	30	28.04					
No	77	71.96					

[a] t-test
[b] Chi-squared test
* PR vs. NEO

Supplementary Table S2.
Clinicopathologic characteristics - Validation cohort

	Complete n = 317		PR n = 251		NEO n = 66		
	n	%	n	%	n	%	*P*-value*
**Age**							
Median (range)	68.00	(28-89)	68.50	(31-89)	66.50	(28-80)	**0.012**[a]
**Gender**							
female	161	50.79	131	52.19	30	45.45	0.33[b]
male	156	49.21	120	47.81	36	54.55	
**pT Stage**							
1	20	6.31	15	5.98	5	7.58	0.866[b]
2	128	40.38	104	41.43	24	36.36	
3	100	31.55	82	32.67	18	27.27	
4	41	12.93	32	12.75	9	13.64	
Unknown	28	8.83	18	7.17	10	15.15	
**pN Stage**							
0	76	23.97	57	22.71	19	28.79	0.159[b]
1	140	44.16	109	43.43	31	46.97	
2	76	23.97	66	26.29	10	15.15	
Unknown	25	7.89	19	7.57	6	9.09	
**cM Stage**							
0	277	87.38	223	88.84	54	81.82	0.126[b]
1	40	12.62	28	11.16	12	18.18	
**UICC Stage**							
I	37	11.67	31	12.35	6	9.09	0.208[b]
II	125	39.43	98	39.04	27	40.91	
III	86	27.13	77	30.68	9	13.64	
IV	26	8.20	21	8.37	5	7.58	
Unknown	43	13.56	24	9.56	0	0.00	
**Neoadjuvant Treatment**							
Yes	66	20.82					
No	233	73.50					
Unknown	18	5.68					

[a] t-test
[b] Chi-squared test
* PR vs. NEO

Supplementary Table S3.
Clinicopathologic characteristics – Validation subcohorts

	Train n = 222		Test n = 95		
	n	%	n	%	*P*-value
**Age**					
Median (range)	68.00	(28-89)	68.00	(45-88)	0.756[a]
**Gender**					
female	107	48.20	54	56.84	0.159[b]
male	115	51.80	41	43.16	
**pT Stage**					
1	16	7.21	4	4.21	0.793[b]
2	89	40.09	39	41.05	
3	71	31.98	29	30.53	
4	28	12.61	13	13.68	
Unknown	18	8.11	10	10.53	
**pN Stage**					
0	55	24.77	21	22.11	0.571[b]
1	101	45.50	39	41.05	
2	50	22.52	26	27.37	
Unknown	16	7.21	9	9.47	
**cM Stage**					
0	197	88.74	80	84.21	0.266[b]
1	25	11.26	15	15.79	
**UICC Stage**					
I	30	13.51	12	12.63	0.540[b]
II	101	45.50	37	38.95	
III	63	28.38	31	32.63	
IV	25	18.02	15	15.79	
Unknown	3	1.35	0	0.00	
**Neoadjuvant Treatment**					
Yes	46	20.72	20	21.05	0.968[b]
No	163	73.42	70	73.68	
Unknown	13	5.86	5	5.26	

[a] t-test

[b] Chi-squared test

Supplementary Table S4.
Clinicopathologic characteristics - Training cohort

	sCD40 low n = 55		sCD40 high n = 167		
	n	%	n	%	*P*-value
**Age**					
Median (range)	66.00	(28-85)	69.00	(31-89)	**0.009**[a]
**Gender**					
female	29	52.73	78	46.71	0438[b]
male	26	47.27	89	53.29	
**pT Stage**					
1	5	9.09	11	6.59	0.69[b]
2	18	32.73	71	42.51	
3	19	34.55	52	31.14	
4	7	12.73	21	12.57	
Unknown	6	10.91	12	7.19	
**pN Stage**					
0	15	27.27	40	23.95	0.126[b]
1	29	52.73	72	43.11	
2	7	12.73	43	25.75	
Unknown	4	7.27	12	7.19	
**cM Stage**					
0	47	85.45	150	89.82	0.374[b]
1	8	14.55	17	10.18	
**UICC Stage**					
I	7	12.73	23	13.77	0.431[b]
II	27	49.09	74	44.31	
III	11	20.00	52	31.14	
IV	8	14.55	17	10.18	
Unknown	2	3.64	0	0.00	
**Neoadjuvant Treatment**					
Yes	16	29.09	30	17.96	0.051[b]
No	34	61.82	129	77.25	
Unknown	5	9.09	8	4.79	

[a] t-test
[b] Chi-squared test

Supplementary Table S5.
Clinicopathologic characteristics - Test cohort

	sCD40 low n = 27		sCD40 high n = 68		
	n	%	n	%	*P*-value
**Age**					
Median (range)	68.00	(45-88)	68.00	(45-83)	0.655[a]
**Gender**					
female	16	59.26	38	55.88	0.764[b]
male	11	40.74	30	44.12	
**pT Stage**					
1	1	3.70	3	4.41	0.867[b]
2	13	48.15	26	38.24	
3	7	25.93	22	32.35	
4	4	14.81	9	13.24	
Unknown	2	7.41	8	11.76	
**pN Stage**					
0	10	37.04	11	16.18	0.098[b]
1	9	33.33	30	44.12	
2	6	22.22	20	29.41	
Unknown	2	7.41	7	10.29	
**cM Stage**					
0	23	85.19	57	83.82	0.87[b]
1	4	14.81	11	16.18	
**UICC Stage**					
I	7	25.93	5	7.35	0.074[b]
II	7	25.93	30	44.12	
III	9	33.33	22	32.35	
IV	4	14.81	11	16.18	
Unknown	0	0.00	0	0.00	
**Neoadjuvant Treatment**					
Yes	5	18.52	15	22.06	0.753[b]
No	20	74.07	50	73.53	
Unknown	2	7.41	3	4.41	

[a] t-test

[b] Chi-squared test

Supplementary Table S6.
Clinicopathologic characteristics of healthy donors (discovery cohort)

	n = 20		
	n (%)	*P*-value*	*P*-value**
**Age**			
Median (range)	64 (52 - 85)	0.38[a]	0.277[a]
**Gender**			
Female	5 (25)	0.079[b]	0.077[b]
Male	15 (75)		

* compared to PDAC cohort (PR)

** compared to PDAC cohort (NEO)

[a] t-test

[b] Chi-squared test

Supplementary Table S7.
Clinicopathologic characteristics of heathy donors (validation cohort)

	**n = 116** n (%)	*P*-value*	*P*-value**
**Age**			
Median (range)	62 (35 - 85)	**<0.001**[a]	**<0.001**[a]
**Gender**			
Female	51 (44)	0.143[b]	0.846[b]
Male	65 (56)		

* compared to PDAC cohort (PR)

** compared to PDAC cohort (NEO)

[a] t-test

[b] Chi-squared test

Supplementary Table S8.
Clinicopathologic characteristics of IPMN

	n = 38<br>n (%)	*P*-value*	*P*-value**
**Age**			
Median (range)	68 (46 - 77)	0.691[a]	0.163[b]
**Gender**			
Female	23 (60.5)	0.337[b]	0.139[b]
Male	13 (34.2)		

* compared to PDAC cohort (PR)

* compared to PDAC cohort (NEO)

[a] t-test

[b] Chi-squared test

Supplementary Table S9.
Contingency table Marker over (high) or below (low) threshold.

		PDAC				
		negative	positive	total		
sCD40	high	24	173	197	Sensitivity:	0.689
	low	92	78	169	Specificity:	0.793
	Total	116	251		Accuracy	0.724
		negative	positive	total		
CA19-9	high	0	182	182	Sensitivity:	0.731
	low	80	65	145	Specificity:	1
	Total	80	249		Accuracy:	0.801
