# Supplementary material for: Serum immune checkpoint profiling identifies soluble CD40 as a biomarker for pancreatic cancer
Source: NPJ Precis Oncol. 2023 Oct 14;7:104. doi: 10.1038/s41698-023-00459-9 (PMC10576756; doi:10.1038/s41698-023-00459-9)
Supplement: Supplementary file 2 — REPORTING SUMMARY [file 41698_2023_459_MOESM2_ESM.pdf]

nature portfolio

Corresponding author(s): Adrian M. Seifert

Last updated by author(s): Sep 11, 2023

Reporting Summary

Nature Portfolio wishes to improve the reproducibility of the work that we publish. This form provides structure for consistency and transparency in reporting. For further information on Nature Portfolio policies, see our Editorial Policies and the Editorial Policy Checklist.

Statistics

For all statistical analyses, confirm that the following items are present in the figure legend, table legend, main text, or Methods section.

n/a	Confirmed	
☐	☒	The exact sample size (*n*) for each experimental group/condition, given as a discrete number and unit of measurement
☐	☒	A statement on whether measurements were taken from distinct samples or whether the same sample was measured repeatedly
☐	☒	The statistical test(s) used AND whether they are one- or two-sided<br>*Only common tests should be described solely by name; describe more complex techniques in the Methods section.*
☐	☒	A description of all covariates tested
☐	☒	A description of any assumptions or corrections, such as tests of normality and adjustment for multiple comparisons
☐	☒	A full description of the statistical parameters including central tendency (e.g. means) or other basic estimates (e.g. regression coefficient) AND variation (e.g. standard deviation) or associated estimates of uncertainty (e.g. confidence intervals)
☐	☒	For null hypothesis testing, the test statistic (e.g. *F*, *t*, *r*) with confidence intervals, effect sizes, degrees of freedom and *P* value noted<br>*Give P values as exact values whenever suitable.*
☒	☐	For Bayesian analysis, information on the choice of priors and Markov chain Monte Carlo settings
☒	☐	For hierarchical and complex designs, identification of the appropriate level for tests and full reporting of outcomes
☐	☒	Estimates of effect sizes (e.g. Cohen's *d*, Pearson's *r*), indicating how they were calculated

Our web collection on statistics for biologists contains articles on many of the points above.

Software and code

Policy information about availability of computer code

Data collection	GEPIA, cBioPortal
Data analysis	GraphPad Prism Ver. 9.0, R statistical software Ver. 4.2.0.

For manuscripts utilizing custom algorithms or software that are central to the research but not yet described in published literature, software must be made available to editors and reviewers. We strongly encourage code deposition in a community repository (e.g. GitHub). See the Nature Portfolio guidelines for submitting code & software for further information.

Data

Policy information about availability of data

All manuscripts must include a data availability statement. This statement should provide the following information, where applicable:

- Accession codes, unique identifiers, or web links for publicly available datasets
- A description of any restrictions on data availability
- For clinical datasets or third party data, please ensure that the statement adheres to our policy

The data supporting this study's findings are available from the corresponding author upon reasonable request.

Research involving human participants, their data, or biological material

Policy information about studies with human participants or human data. See also policy information about sex, gender (identity/presentation), and sexual orientation and race, ethnicity and racism.

Reporting on sex and gender	The term gender was used in this study, since data where obtained via the patients' medical files. No significant differences was observed between different cohorts regarding gender. Thus, gender was not included into more profound analyses.
Reporting on race, ethnicity, or other socially relevant groupings	No analyses based on race, ethnicity or other socially relevant groupings were performed.
Population characteristics	See section "Behavioural & social sciences study design"
Recruitment	Patients with available serum samples were included in a sequentially manner regarding time of treatment.
Ethics oversight	Ethics Committee of the TU Dresden (Ref-Nr.: 446112017) and the Ethics Committee Göttingen (Ref-Nr.: 24/4/03)

Note that full information on the approval of the study protocol must also be provided in the manuscript.

Field-specific reporting

Please select the one below that is the best fit for your research. If you are not sure, read the appropriate sections before making your selection.

☒ Life sciences ☐ Behavioural & social sciences ☐ Ecological, evolutionary & environmental sciences

For a reference copy of the document with all sections, see nature.com/documents/nr-reporting-summary-flat.pdf

Life sciences study design

All studies must disclose on these points even when the disclosure is negative.

Sample size	Our study is purely exploratory and not a randomized clinical trial. We did not intend to test any formal statistical hypotheses about the efficacy of a medicinal product or treatment and rather focused on investigating the variability in the data . Overall, the sample size calculations to achieve either a nominal test power in hypothesis testing or a prespecified estimate precision are not mandatory for exploratory studies.
Data exclusions	Patients were selected for analysation when there was a histologically confirmed PDAC. Patients were excluded for analyzation, when secondary carcinoma or conflicting histology was found.
Replication	Results were verified using an additional method, a validation cohort and the hold-out method for validating the results of cut-off optimization.
Randomization	Healthy donors or patients suffering from IPMN were used as controls. Randomization was only used for the hold-out method. Sample-function of the Rcpp package in R was used.
Blinding	Our study was exploratory, no blinding was needed.

Reporting for specific materials, systems and methods

We require information from authors about some types of materials, experimental systems and methods used in many studies. Here, indicate whether each material, system or method listed is relevant to your study. If you are not sure if a list item applies to your research, read the appropriate section before selecting a response.

Materials & experimental systems

n/a	Involved in the study	
☒	☐	Antibodies
☒	☐	Eukaryotic cell lines
☒	☐	Palaeontology and archaeology
☒	☐	Animals and other organisms
☐	☒	Clinical data
☒	☐	Dual use research of concern
☒	☐	Plants

Methods

n/a	Involved in the study	
☒	☐	ChIP-seq
☒	☐	Flow cytometry
☒	☐	MRI-based neuroimaging

Clinical data

Policy information about clinical studies

All manuscripts should comply with the ICMJE guidelines for publication of clinical research and a completed CONSORT checklist must be included with all submissions.

Clinical trial registration	NA
Study protocol	Our study was exploratory. Study design is stated in the mansucript
Data collection	Patient samples collected at the Clinic of General, Visceral and Pediatric Surgery, University Medical Center Göttingen (Göttingen, Germany) and the Department of Visceral, Thoracic and Vascular Surgery at the University Hospital Carl Gustav Carus (Dresden, Germany) from 2005 - 2019 were used.
Outcomes	Our study was exploratory. No outcome was defined.
